# Does the preoperative platelet-to-lymphocyte ratio and neutrophil-to-lymphocyte ratio predict morbidity after gastrectomy for gastric cancer?

**DOI:** 10.1186/s40779-020-00234-y

**Published:** 2020-02-29

**Authors:** İbrahim Mungan, Çilem Bayındır Dicle, Şerife Bektaş, Sema Sarı, Serdar Yamanyar, Mine Çavuş, Sema Turan, Erdal Birol Bostancı

**Affiliations:** 1 Department of Intensive Care Unit, Ankara Eğitim ve Araştırma Şehir Hastanesi, 06800 Ankara, PA Turkey; 2 Department of Gastrointestinal Surgery, Ankara Eğitim ve Araştırma Şehir Hastanesi, 06800 Ankara, PA Turkey

**Keywords:** Preoperative, Platelet-to-lymphocyte ratio, Neutrophil-to-lymphocyte ratio, Morbidity, Gastrectomy, Stomach cancer

## Abstract

**Background:**

Gastric cancer is the 2nd most common cause of cancer-related deaths, and the morbidity rate after surgery is reported to be as high as 46%. The estimation of possible complications, morbidity, and mortality and the ability to specify patients at high risk have become substantial for an intimate follow-up and for proper management in the intensive care unit. This study aimed to determine the prognostic value of the preoperative platelet-to-lymphocyte ratio (PLR) and neutrophil-to-lymphocyte ratio (NLR) and their relations with clinical outcomes and complications after gastrectomy for gastric cancer.

**Methods:**

This single-center, retrospective cohort study evaluated the data of 292 patients who underwent gastrectomy with curative intent between January 2015 and June 2018 in a tertiary state hospital in Ankara, Turkey. A receiver operating characteristic curve was generated to evaluate the ability of laboratory values to predict clinically relevant postoperative complications. The area under the curve was computed to compare the predictive power of the NLR and PLR. Then, the cutoff points were selected as the stratifying values for the PLR and NLR.

**Results:**

The area under the curve values of the PLR (0.60, 95% CI 0.542–0.657) and NLR (0.556, 95% CI 0.497–0.614) were larger than those of the other preoperative laboratory values. For the PLR, the diagnostic sensitivity and specificity were 50.00 and 72.22%, respectively, whereas for the NLR, the diagnostic sensitivity and specificity were 37.50 and 80.16%, respectively. The PLR was related to morbidity, whereas the relation of the NLR with mortality was more prominent. This study demonstrated that the PLR and NLR may predict mortality and morbidity via the Clavien-Dindo classification in gastric cancer patients. The variable was grade ≥ 3 in the Clavien-Dindo classification, including complications requiring surgical or endoscopic interventions, life-threatening complications, and death. Both the PLR and NLR differed significantly according to Clavien-Dindo grade ≥ 3. In this analysis, the PLR was related to morbidity, while the NLR relation with mortality was more intense.

**Conclusion:**

Based on the results of the study, the PLR and NLR could be used as independent predictive factors for mortality and morbidity in patients with gastric cancer.

## Background

Gastric cancer (GC), which is the 2nd most common cause of cancer-related deaths, usually presents with nonspecific symptoms and is diagnosed in the late stages [1]. The mortality rate per case is reported to be as high as 70%, whereas the morbidity rate after surgery is reported to be as high as 46% [2]. Total or subtotal gastrectomy with lymphadenectomy is the foundation of surgical interventions for GC, and it is the only one with curative potential.

Despite improvements in surgical techniques, gastrectomy still has postoperative complication risks, such as anastomotic leaks, as in all surgical treatment modalities, and these complications lead to increased morbidity and mortality rates. Increased morbidity causes longer hospital stays, increased hospital costs, the postponement of chemotherapy and diminished quality of life [3]. In recent years, it has been shown that wound healing and infection control, especially in the anastomoses line, mainly determine the morbidity rate in surgical patients, and inflammatory, immunological or nutritional indices are used to predict mortality and morbidity [4]. The Clavien-Dindo (CD) classification is used to grade postoperative complications after gastrectomy, and recent studies have evaluated the prognostic power of numerous indices via the CD classification [5].

Although the clinicopathological prognostic indicators of GC, which are the American Joint Committee on Cancer (AJCC) stage and size, histological type and grade, and lymphovascular and perineural invasion, are the most significant prognostic factors, a simple and easily provided predictive index requirement has been emphasized in recent studies. In addition, it was also claimed that patients with identical clinicopathological properties did not experience homogenous clinical outcomes [6]. The estimation of possible complications, morbidity, and mortality and the ability to specify patients at high risk have become substantial for an intimate follow-up and for proper management in the intensive care unit (ICU) [7].

Platelets, lymphocytes, and neutrophils are easily detected on a routine blood count, and each of them plays an important part in the inflammatory and anti-inflammatory processes, immune response, and coagulation status, which are related to tumor progression and prognosis in various solid cancers. Tumor growth leads to the increased production of inflammatory cytokines and growth factors (mainly IL-1훽, IL-3, IL-6, IL-11, IL-23, and TNF-훼), and this perpetual process ensures immortality. These promoting factors are also important for angiogenesis and hematopoiesis, which explains the increase in blood cell types in cancerous diseases [8]. A poor prognosis is claimed to be related to an increased platelet count, younger platelets in the circulation, and the imbalance between lymphocytes and neutrophils, especially in gastric, kidney and lung cancers [8, 9]. The platelet-to lymphocyte ratio (PLR), neutrophil-to-lymphocyte ratio (NLR), and platelet count have been offered as inflammatory and prognostic indicators with increasing evidence not only in solid cancers but also in cardiovascular disease and renal failure [10, 11].

The study aimed to determine the prognostic value of the preoperative PLR and NLR and their relations with clinical outcomes and complications after gastrectomy for gastric cancer.

## Methods

This single-center, retrospective cohort study evaluated the data of patients who underwent gastrectomy with curative intent between January 2015 and June 2018 in a tertiary state hospital in Ankara, Turkey. The inclusion criteria were the patients who were histologically proven to have GC and underwent elective surgery. The exclusion criteria were the patients with incomplete clinicopathological or laboratory data and patients with a history of other malignancies. The emergent surgery, acute infection, systemic inflammation, autoimmune disturbances, and hematologic disorders were the other exclusion criteria. A total of 196 male and 96 female patients aged 24 to 86 years (mean age 61.1 years) were enlisted in the final assessment.

Although the study was in the category of noninterventional clinical research with its retrospective nature, we did apply for ethics committee approval. The ethics committee approved the study (No: 72300690–799) and formal consent in addition to what the patients had given prior to hospitalization was waived. This research complied with the principles outlined in the Helsinki Declaration of 1975, as revised in 2008.

### Data acquisition

Detailed clinicopathological and demographic data, including patient age, sex, tumor location, histological grade, clinical TNM stage [in accordance with the TNM staging system of the American Joint Committee on Cancer (AJCC 7th ed., 2010)] [12], the extent of gastrectomy, the presence of distant metastases and the outcomes (such as mortality and anastomotic leaks) as well as complete blood count values (including neutrophils, platelets, and lymphocytes) in the preoperative period, preferably the day before surgery, were collected retrospectively from the institutional database. These data were imported into a spreadsheet (Microsoft Excel 2013, Microsoft Corporation, Redmond, WA, USA) and used for the calculation of prognostic indices. To forestall the variability in the data collection, all values were reviewed by the author of the study.

To calculate the NLR and PLR, the formulas were used described below:

$$ \mathrm{NLR}=\left(\mathrm{absolute}\ \mathrm{neutrophil}\ \mathrm{count}\right)/\left(\mathrm{total}\ \mathrm{lymphocyte}\ \mathrm{count}\right);\mathrm{and}\ \mathrm{PLR}=\left(\mathrm{total}\ \mathrm{lymphocyte}\ \mathrm{count}\right)/\left(\mathrm{total}\ \mathrm{platelet}\ \mathrm{count}\right)\times 100. $$

### Statistical analysis

SPSS for Windows (version 20.0, SPSS, Inc., Chicago, IL, USA) and MedCalc 15.8 software (MedCalc, Ostend, Belgium) were used for statistical analyses. The continuous variables are presented as the mean ± standard deviation (SD), while nominal variables are presented as the total number and percentage.

First, the variables were evaluated with the one-sample Kolmogorov-Smirnov test as a normality test, and the results showed that asymp. Sig. (2-tailed) levels were ≤ 0.05. Therefore, nonparametric tests were used.

As the second step, a receiver operating characteristic (ROC) curve was generated to evaluate the ability of laboratory values to predict clinically relevant postoperative complications (≥grade 3 according to the CD classification). These complications were grade 3 - complications requiring surgical, endoscopic or radiological intervention; grade 4 - life-threatening complications (including central nervous system complications); and grade 5 - mortality.

In this analysis, the area under the curve (AUC) was computed to compare the predictive power of the laboratory values, mainly the NLR and PLR. In addition, the cutoff points computed with the maximal Youden index were selected as the stratifying values for the PLR and NLR. After that, the study population was grouped into two groups according to these cutoff points for each prominent variable. One group was representing those below the cutoff point, while the other group was representing those above the cutoff point.

At the final stage, categorical and continuous variables were evaluated by the Mann-Whitney *U* test and Spearman’s Rho test where appropriate. In all analyses, the *p-*value of less than 0.05 was considered statistically significant.

## Results

**Fig. 1:**
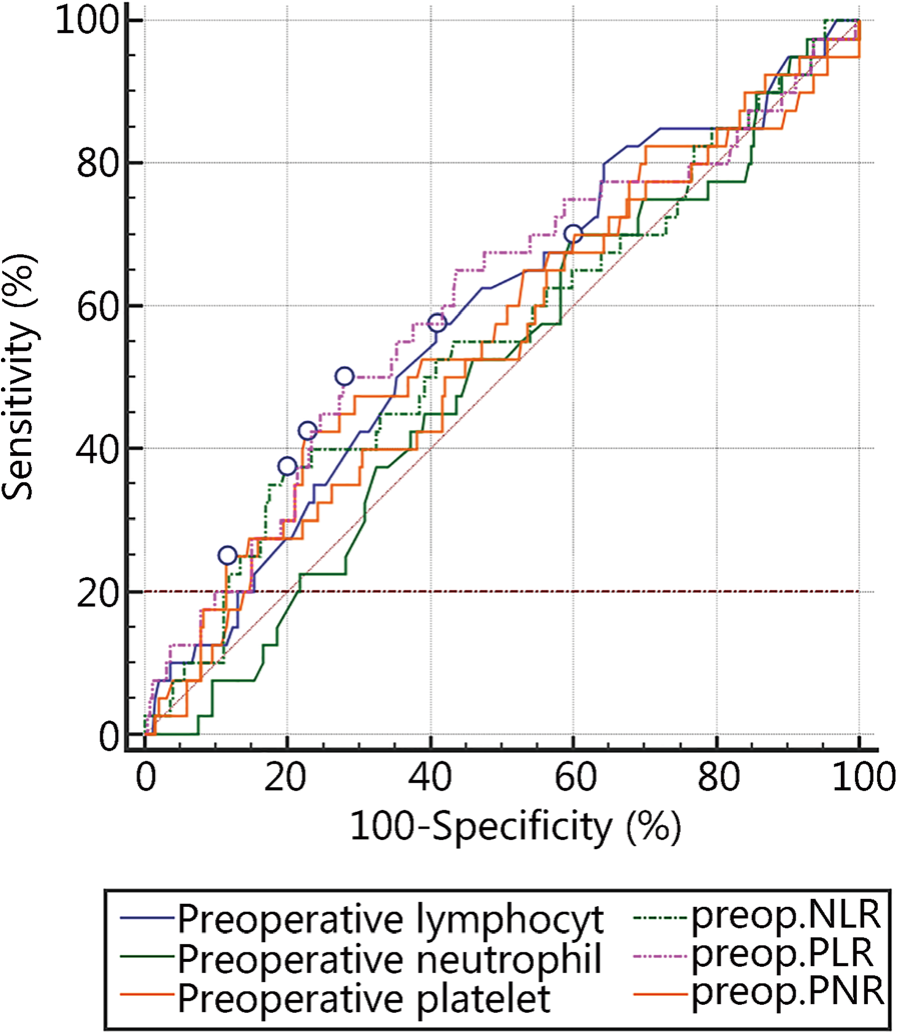
The predictive powers of different laboratory variables were compared with the AUC values according to the postoperative complications grade ≥ 3 CD classification. AUC. Area under the curve; CD. Clavien-Dindo; NLR. Neutrophil-to-lymphocyte ratio; PLR. Platelet-to-lymphocyte ratio; PNR. Platelet-to-neutrophil ratio; preop. Preoperative

As explained above, the study evaluated the predictive power of different variables on the aforementioned postoperative complications, and the results are summarized in Table 1 and Fig. 1.

**Table 1:** Comparison of the AUCs of preoperative laboratory values according to the postoperative complications grade ≥ 3 CD classification

Item	AUC	SE	95% CI
Preoperative lymphocyte value	0.585	0.0488	0.526–0.642
Preoperative neutrophil value	0.504	0.0476	0.446–0.563
Preoperative platelet value	0.569	0.0516	0.510–0.626
Preoperative mean platelet volume	0.511	0.0535	0.452–0.569
Preoperative platelet distribution width	0.507	0.0515	0.448–0.566
Preoperative PLR	0.600	0.0517	0.542–0.657
Preoperative NLR	0.556	0.0521	0.497–0.614
Preoperative PNR	0.546	0.0508	0.487–0.604

*AUC* area under the curve, *CI* confidence interval, *NLR* neutrophil-to-lymphocyte ratio, *PLR* platelet-to-lymphocyte ratio, *PNR* platelet-to-neutrophil ratio, *SE* standard error

As shown in Table 1, the AUC values of the PLR (0.60, 95% CI 0.542–0.657) and NLR (0.556, 95% CI 0.497–0.614) were larger than those of the other preoperative laboratory values. The AUC values of the platelet count and lymphocyte count were comparable to but smaller than that of the PLR, and their discriminative power was worse. The AUC value of the PLR or NLR could be judged unsatisfactory, while the findings indicated that the PLR and NLR had stronger predictive power compared with the other preoperative laboratory values. The aim was also to determine the relations between the PLR and NLR with clinical outcomes and complications, so the study used the ROC curves to determine the cutoff points of the PLR and NLR. The cutoff points were computed with the maximal Youden index and compared in Table 2.

**Table 2:** AUCs, statistical findings associated criterion, and maximum sensitivity and specificity of the PLR and NLR with the optimal cutoff points

Results	PLR	NLR
AUC	0.60	0.556
SE	0.05	0.0521
95% CI	0.542–0.657	0.497–0.614
z statistic	1.937	1.084
Youden index	0.2222	0.1766
Associated criterion	≤0.55	> 3.92
Sensitivity (%)	50.00	37.50
Specificity (%)	72.22	80.16

*AUC* area under the curve, *CI* confidence interval, *NLR* neutrophil-to-lymphocyte ratio, *PLR* platelet-to-lymphocyte ratio, *SE* standard error.

After the completion of this assessment, 0.55 and 3.92 were selected as the stratifying values for the PLR and NLR, respectively. For the PLR, the diagnostic sensitivity and specificity were 50.00 and 72.22%, respectively, whereas for the NLR, the diagnostic sensitivity and specificity were 37.50 and 80.16%, respectively. Among the enlisted patients, 30.5% of patients (*n =* 89) had PLR values ≤0.55, and 22.6% of patients (*n* = 66) had NLR values > 3.92.

In this study, 292 patients with GC enlisted, and 196 (67.1%) were male. As shown in Table 3, the demographic and clinicopathological variables were compared according to the PLR and NLR values, and no difference was detected with respect to sex, while the difference was statistically significant with respect to age.

**Table 3:** Demographic and clinicopathological variables compared according to the PLR and NLR values

Item	All (*N =* 292)	PLR ≤ 0.55 (*n =* 89)	PLR > 0.55 (*n =* 203)	*P value*^*+*^	NLR ≤ 3.92 (*n =* 226)	NLR > 3.92 (*n =* 66)	*P value*^*+*^
Age (years, mean ± SD)	61.1 ± 11.3	64.6 ± 11.0	59.6 ± 11.1	< 0.001^*^	60.3 ± 11.2	64 ± 11.2	0.033^*^
Sex (Male) [*n*(%)]	196 (67.1)	65 (73.0)	131 (64.5)	0.156	147 (65.0)	49 (74.2)	0.163
AJCC stage				0.01^3*^			0.004^*^
Stage 1[*n*(%)]	70 (24.0)	15 (16.9)	55 (27.1)		62 (27.4)	8 (12.1)	
Stage 2[*n*(%)]	82 (28.1)	25 (28.1)	57 (28.1)		64 (28.3)	18 (27.3)	
Stage 3[*n*(%)]	107 (36.6)	32 (36.0)	75 (36.9)		78 (34.5)	29 (43.9)	
Stage 4[*n*(%)]	33 (11.3)	17 (19.1)	16 (7.9)		22 (9.7)	11 (16.7)	
Tumor extent (size)				0.002^*^			0.001^*^
1[*n*(%)]	62 (21.2)	11 (12.4)	51 (25.1)		57 (25.2)	5 (7.6)	
2[*n*(%)]	40 (13.7)	12 (13.5)	28 (13.8)		32 (14.2)	8 (12.1)	
3[*n*(%)]	164 (56.2)	52 (58.4)	112 (55.2)		120 (53.1)	44 (66.7)	
4[*n*(%)]	26 (8.9)	14 (15.7)	12 (5.9)		17 (7.5)	9 (13.6)	
Lymph node count				0.794			0.240
0[*n*(%)]	83 (28.4)	24 (27.0)	59 (29.1)		68 (30.1)	15 (22.7)	
1[*n*(%)]	60 (20.5)	18 (20.2)	42 (20.7)		48 (21.2)	12 (18.2)	
2[*n*(%)]	50 (17.1)	17 (19.1)	33 (16.3)		35 (15.5)	15 (22.7)	
3[*n*(%)]	99 (33.9)	30 (33.7)	69 (34.0)		75 (33.2)	24 (36.4)	
Distant metastases [*n*(%)]	33 (11.3)	17 (19.1)	16 (7.9)	0.005^*^	22(9.7)	11 (16.7)	0.118
Location of the tumor				0.259			0.840
Cardia/ fundus [*n*(%)]	54 (18.5)	24 (27.0)	30 (14.8)		39 (17.3)	15 (22.7)	
Body [*n*(%)]	194 (66.4)	49 (55.1)	145 (71.4)		155 (68.6)	39 (59.1)	
Antrum/ pylorus [*n*(%)]	44 (15.1)	16 (18.0)	28 (13.8)		32 (14.2)	12 (18.2)	
Total gastrectomy [*n*(%)]	152 (52.1)	53(59.6)	99 (48.8)	0.090	116 (51.3)	36 (54.5)	0.647
Size of the tumor> 3 cm [*n*(%)]	221 (75.7)	71 (79.8)	150 (73.9)	0.282	166 (73.5)	55 (83.3)	0.100
Histological grade				0.542			0.852
Well differentiated [*n*(%)]	71 (24.3)	20 (22.5)	51 (25.1)		55 (24.3)	16 (24.2)	
Moderately differentiated [*n*(%)]	103 (35.3)	36 (40.4)	67 (33.0)		81 (35.8)	22 (33.3)	
Poorly differentiated [*n*(%)]	77 (26.4)	25 (28.1)	52 (25.6)		58 (25.7)	19 (28.8)	
Signet ring [*n*(%)]	41 (14.0)	8 (9.0)	33 (16.3)		32 (14.2)	9 (13.6)	
Lymphatic invasion [*n*(%)]	211 (72.3)	66 (74.2)	145 (71.4)	0.633	155 (68.6)	56 (84.8)	0.009^*^
Perineural invasion [*n*(%)]	161 (55.1)	50 (56.2)	111 (54.7)	0.737	118 (52.2)	43 (65.2)	0.047^*^
Vascular invasion [*n*(%)]	170 (58.2)	54 (60.7)	116 (57.1)	0.575	126 (55.8)	44 (66.7)	0.115

*P value*^*+*^ were calculated for the comparison of groups based on the NLR and PLR values by statistical analysis; +Determined by the Mann-Whitney U test or Spearman’s rho test; *. *P value <* 0.05; *AJCC* American Joint Committee on Cancer, *PLR* platelet-to-lymphocyte ratio, *NLR* neutrophil-to-lymphocyte ratio

In total, 107 (36.6%) patients were in stage III according to the AJCC classification, and this variable and the extent (size) of the tumor (T) differed significantly between groups. Interestingly, the lymph node (N) status did not differ significantly. Of the patients, 75.7% had a tumor size ≥3 cm. The size of the tumor, location of the tumor, extent of gastrectomy and histological grade of the tumor as variables were not significantly different. Distant metastasis was different between groups based on the PLR, whereas lymphatic invasion and perineural invasion showed a significant difference based on the NLR.

The clinical outcomes and postoperative complications graded with the CD classification are shown in Table 4. If the study population was grouped according to the PLR (cutoff point 0.55), the length of stay (LOS) in the hospital and in the ICU, anastomotic leaks, postoperative complications and CD ≥ grade 3 were significantly different between groups. The mortality rate did not differ between groups. When the groups were divided according to the NLR value (cutoff point 3.92), the LOS in the hospital, CD grade ≥ 3 and especially mortality as variables differed significantly. This difference was not detected with respect to the LOS in the ICU, anastomotic leaks or postoperative complications as variables.

**Table 4:** Outcomes and clinical variables between the groups.

Item	All (*N =* 292)	PLR ≤ 0.55 (*n =* 89)	PLR > 0.55 (*n =* 203)	*P value*^*+*^	NLR ≤ 3.92 (*n =* 226)	NLR > 3.92 (*n =* 66)	*P value*^*+*^
LOS in the hospital (days, mean ± SD)	16.0 ± 11.9	18.3 ± 12.9	15.0 ± 11.4	0.018^*^	15.5 ± 12.0	17.8 ± 11.7	0.027^*^
LOS in the ICU (days, mean ± SD)	6.3 ± 9.5	8.6 ± 10.9	5.4 ± 8.7	0.002^*^	5.9 ± 9.6	7.7 ± 9.1	0.062
Anastomotic leaks [*n*(%)]	36 (12.3)	18 (20.2)	18 (8.9)	0.006^*^	24 (10.6)	12 (18.2)	0.101
Complications				0.008^*^			0.671
None [*n*(%)]	266 (91.1)	75 (84.3)	191 (94.1)		207 (91.6)	59 (89.4)	
Cardiac [*n*(%)]	10 (3.4)	6 (6.7)	4 (2.0)		5 (2.2)	5 (7.6)	
Respiratory [*n*(%)]	9 (3.1)	6 (6.7)	3 (1.5)		7 (3.1)	2 (3.0)	
Infectious [*n*(%)]	2 (0.7)	0	2 (1.0)		2 (0.9)	0	
Renal [*n*(%)]	5 (1.7)	2 (2.2)	3 (1.5)		5 (2.2)	0	
Clavien-Dindo grade ≥ 3[*n*(%)]	40(13.7)	19 (21.3)	21 (10.3)	0.012*	25 (11.1)	15 (22.7)	0.015^*^
In-hospital mortality [*n*(%)]	9 (3.1)	5 (5.6)	4 (2.0)	0.098	2 (0.9)	7 (10.6)	< 0.001^*^

*P value*^*+*^ were calculated for the comparison of groups based on the NLR and PLR values by statistical analysis; +Determined by the Mann-Whitney U test or Spearman’s rho test; *. *P value <* 0.05; *ICU* intensive care unit, *LOS* length of stay, *NLR* neutrophil-to-lymphocyte ratio, *PLR* platelet-to-lymphocyte ratio

## Discussion

Inflammation and tumor growth are dependent factors, and increasing numbers of studies have elucidated the role of systemic inflammatory response mediators in various solid cancers [13, 14]. Among them, GC is one of the popular foci of these investigations that evaluated cancer-related inflammation and possible predictors in the preoperative period [6–8]. The decrease in lymphocyte count leads to depression of the immune response and cytotoxic destruction, whereas increased platelets and neutrophils induce tumor progression and angiogenesis [3]. Hence, this study was implemented to investigate the predictive power and the relations of the PLR and NLR with morbidity, mainly Clavien-Dindo grade ≥ 3, and mortality in patients with GC. Although the exact mechanism by which the PLR or NLR influences outcomes in GC patients is not clear, the NLR and PLR are widely used and easily obtained at very low costs [6, 11].

This study demonstrated that the PLR and NLR may predict mortality and morbidity via the CD classification in GC patients. The grade ≥ 3 in CD was utilized as a variable, and this variable included complications requiring surgical or endoscopic interventions, life-threatening complications, and death. Both the PLR and NLR differed significantly according to Clavien-Dindo grade ≥ 3. The cutoff values were also employed, which were determined by ROC curves and the Youden index, to investigate the relations of the PLR and NLR with each of the variables, such as mortality, anastomotic leaks, postoperative complications, LOS in the hospital and LOS in the ICU. In the analysis, the PLR was related to morbidity, while the NLR relation with mortality was more intense. This result is similar to that from a meta-analysis that showed that a high NLR correlated with mortality [15].

Various studies have recommended different cutoff points for the NLR (ranging between 3 and 5) and PLR (ranging between 0.66 and 0.44) [3, 7, 13]. In the literature, different calculations have been proposed for the PLR, and this study used a relatively less common method (as described in the study by Inaoka et al.) [3]. In this study, the cutoff point for the NLR was 3.92 (sensitivity 37.50%, specificity 80.16%), and the cutoff point for the PLR was 0.55 (sensitivity 50.00%, specificity 72.22%). In particular, the specificities of the cutoff points of the NLR and PLR in the results were high; however, the sensitivities decreased the power of the analysis.

The various calculation methods used for the PLR and NLR and dissimilar, non-standardized study populations might have led to this difference. Different cohort studies with different aged populations and clinical situations should be performed and meta-analyzed to determine the best cutoff value for predicting morbidity in gastric cancer.

Shimada et al. [16] suggested that older age was an independent risk factor for the NLR, and in this study, the findings were similar. The correlations of the AJCC stage and T with the PLR and NLR variables were statistically significant, similar to the findings of Sun et al. [17], and this increases the value of the study because of the difficulty of the preoperative estimation of tumor stage and lymph node invasion. This association was not detected with histological grade, and it was partially correlated with invasion (lymphatic, perineural and vascular invasion). These correlations are important, especially in unresectable GC patients.

Kim et al. [18] declared the predictive power of the NLR and PLR in GC patients. Two other meta-analyses investigated the correlations of the PLR with clinicopathologic characteristics, morbidity, and mortality in colorectal cancer patients [19, 20]. Growing data and studies about inflammatory markers and prognosis in GC patients indicate the use of these markers as predictors [3, 6–8].

This study was based on retrospective data of GC patients in a single center with a small sample size, and this was the main limitation. The second limitation was that only the laboratory values in the preoperative period were evaluated. The changes in these values or the effects of any change during the ICU period could not be assessed.

## Conclusions

We must admit that prospective and indiscriminate studies are required to confirm the findings, but based on the results of this study, the PLR and NLR could be used as independent predictive factors for mortality and morbidity in patients with GC.

## Data Availability

The datasets used and/or analyzed during the current study are available from the corresponding author on reasonable request.

## Abbreviations

AJCC: American Joint Committee on Cancer
AUC: Area under the curve
CD classification: Clavien-Dindo classification
CI: Confidence interval
GC: Gastric cancer
ICU: Intensive care unit
LOS: Length of stay
Nl: Lymph node status
NLR: Neutrophil-to-lymphocyte ratio
PLR: Platelet-to lymphocyte ratio
PNR: Platelet-to-neutrophil ratio
ROC: Receiver operating characteristic
SE: Standard error
T: The extent (size) of the tumor
