# Correction to: Does the preoperative platelet-tolymphocyte ratio and neutrophil-tolymphocyte ratio predict morbidity after gastrectomy for gastric cancer?

**DOI:** 10.1186/s40779-020-00242-y

**Published:** 2020-03-23

**Authors:** İbrahim Mungan, Çilem Bayındır Dicle, Şerife Bektaş, Sema Sarı, Serdar Yamanyar, Mine Çavuş, Sema Turan, Erdal Birol Bostancı

**Affiliations:** 1 Department of Intensive Care Unit, Ankara Eğitim ve Araştırma Şehir Hastanesi, 06800 Ankara, PA Turkey; 2 Department of Gastrointestinal Surgery, Ankara Eğitim ve Araştırma Şehir Hastanesi, 06800 Ankara, PA Turkey

**Correction: Mil Med Res (2020) 7:9**

**https://doi.org/10.1186/s40779-020-00234-y**

In the original publication of this article [1] there are two garbled codes in the second sentence, the fourth paragraph of the Background section. The correct sentence should be: Tumor growth leads to the increased production of inflammatory cytokines and growth factors (mainly IL-1*β*, IL-3, IL-6, IL-11, IL-23, and TNF-*α*), and this perpetual process ensures immortality. These promoting factors are also important for angiogenesis and hematopoiesis, which explains the increase in blood cell types in cancerous diseases. The original publication has been corrected.

## References

[1] Mungan İ (2020). Does the preoperative platelet-tolymphocyte ratio and neutrophil-tolymphocyte ratio predict morbidity after gastrectomy for gastric cancer?. Mil Med Res.

